# Rice Stress-Resistant SNP Database

**DOI:** 10.1186/s12284-019-0356-0

**Published:** 2019-12-23

**Authors:** Samuel Tareke Woldegiorgis, Shaobo Wang, Yiruo He, Zhenhua Xu, Lijuan Chen, Huan Tao, Yu Zhang, Yang Zou, Andrew Harrison, Lina Zhang, Yufang Ai, Wei Liu, Huaqin He

**Affiliations:** 1 College of Life Sciences, Fujian Agriculture and Forestry University, Fuzhou, 350002 China; 2 School of Science and Engineering, The Chinese University of Hong Kong, Shenzhen, 518172 China; 3 Department of Mathematical Sciences, University of Essex, Colchester, UK

**Keywords:** SNP, Biotic stress, Abiotic stress, Database

## Abstract

**Background:**

Rice (*Oryza sativa* L.) yield is limited inherently by environmental stresses, including biotic and abiotic stresses. Thus, it is of great importance to perform in-depth explorations on the genes that are closely associated with the stress-resistant traits in rice. The existing rice SNP databases have made considerable contributions to rice genomic variation information but none of them have a particular focus on integrating stress-resistant variation and related phenotype data into one web resource.

**Results:**

Rice Stress-Resistant SNP database (http://bioinformatics.fafu.edu.cn/RSRS) mainly focuses on SNPs specific to biotic and abiotic stress-resistant ability in rice, and presents them in a unified web resource platform. The Rice Stress-Resistant SNP (RSRS) database contains over 9.5 million stress-resistant SNPs and 797 stress-resistant candidate genes in rice, which were detected from more than 400 stress-resistant rice varieties. We incorporated the SNPs function, genome annotation and phenotype information into this database. Besides, the database has a user-friendly web interface for users to query, browse and visualize a specific SNP efficiently. RSRS database allows users to query the SNP information and their relevant annotations for individual variety or more varieties. The search results can be visualized graphically in a genome browser or displayed in formatted tables. Users can also align SNPs between two or more rice accessions.

**Conclusion:**

RSRS database shows great utility for scientists to further characterize the function of variants related to environmental stress-resistant ability in rice.

## Background

Rice (*Oryza sativa* L.) is one of the significant cereal crops, feeding a large number of worldwide populations. It is also one of the well-studied model organisms for plant research. The need to breed robust and high-productivity rice varieties is more critical than ever due to increasingly adverse environmental conditions and scarce natural resources. Rice yield is limited inherently by environmental stresses, including biotic and abiotic stresses. Rice breeders try to limit yield losses from environmental stresses by incorporating resistant genes and developing more climatically resilient cultivars. Thus, it is of great importance to perform in-depth explorations on the genes that are closely associated with the stress-resistant traits in rice.

The recent advances in rice genome biology has generated a tremendous amount of valuable data, including a high-quality reference genome provided by the MSU Rice Genome Annotation Project (Ouyang et al. 2007) and the International Rice Genome Sequencing Project’s (IRGSP) RAPdb (Kawahara et al. 2013; Sakai et al. 2013) and genome re-sequencing data of 3010 rice accessions in the rice germplasm core collection (Li et al. 2014). The achievement of those rice genome datasets and the discovery of large numbers of single nucleotide polymorphisms (SNPs) in genome-scale sequencing initiatives opening new doors into the study of the genome-wide distribution of diversity, the design of molecular markers for genetic mapping of quantitative trait loci (QTLs) or genes, and the evolutionary dynamics of rice genomes (Feltus et al. 2004).

The optimal management of the huge amount of biological information requires the implementation of dedicated bioinformatics facilities. For this reason, a number of integrative databases and web resources for rice genomic variations had been created and curated. IC4R Rice Variation Database collected almost all the available genome re-sequenced data for different rice varieties, identified about 18 M SNPs and curated the SNP effects by integrating available genotype-to-phenotype association results (Zhang et al. 2016). RiceVarMap is another comprehensive database for rice genomic variation and its functional annotation (Zhao et al. 2015). Rice SNP-Seek Database (Alexandrov et al. 2015; Mansueto et al. 2017), which resulted from 3000 Rice Genomes Project, provided genotype, phenotype and variety information for rice. SNP haplotype database is a haplotype map database which curated validated SNP information collections from around the world with a specific focus on Japanese rice collections (Yonemaru et al. 2014). Most of these databases focused mainly on overall variation from a vast set of rice varieties. On the other hand, some databases have been designed with particular focus on specialized rice genomic data, such as OryzaGenome (Ohyanagi et al. 2016), a genome variation database for 21 wild *Oryza* species and 446 *O. rufipogon* varieties. STIFDB2 (Stress Responsive Transcription Factor Database), is a database for transcription factors and consensus binding sites of stress-responsive genes in Arabidopsis and rice (Naika et al. 2013), while PRGdb is a bioinformatics platform for plant resistant gene analysis (Sanseverino et al. 2010; Osuna-Cruz et al. 2018).

Although the existing related databases have made considerable contributions to rice genomic variation information, none of them presented the rice genomic variations based on special traits such as rice stress resistance/susceptibility. Additionally, the existing SNP databases have not yet incorporated the variants for some of the rice stress resistant varieties. On the other hand, the existing rice stress phenotype databases provide only the phenotypic data of the stress resistant/varieties. Therefore, the we reason the importance of developing a dedicated database for rice stress resistant SNPs, which accommodates the phenotypic data and genomic variations of rice stress resistant varieties in one unified bioinformatics platform would greatly benefit rice researchers. The availability of such an interactive database to explore genomic variability in rice stress-resistant varieties will facilitate research studies, provide scientists a handy tool to access information regarding genetic variants of abiotic and biotic stress resistant rice varieties and help them to identify potential candidate genes for rice stress-resistance.

In this paper, we present a database, the RSRS database (http://bioinformatics.fafu.edu.cn/ RSRS), which is a specifically designed database to present the genomic variation of abiotic and biotic stresses-resistant rice varieties. We first collected the genomic re-sequencing data of over 400 rice varieties with different resistant ability to different biotic and abiotic stresses from previous researches, detected the SNPs in these rice variety genomes, and then screened the stress-resistant SNPs in the resistant rice varieties and catalogued these SNPs in a database system.

## Materials and Methods

### Data Collection and Organization

**Fig. 1:**
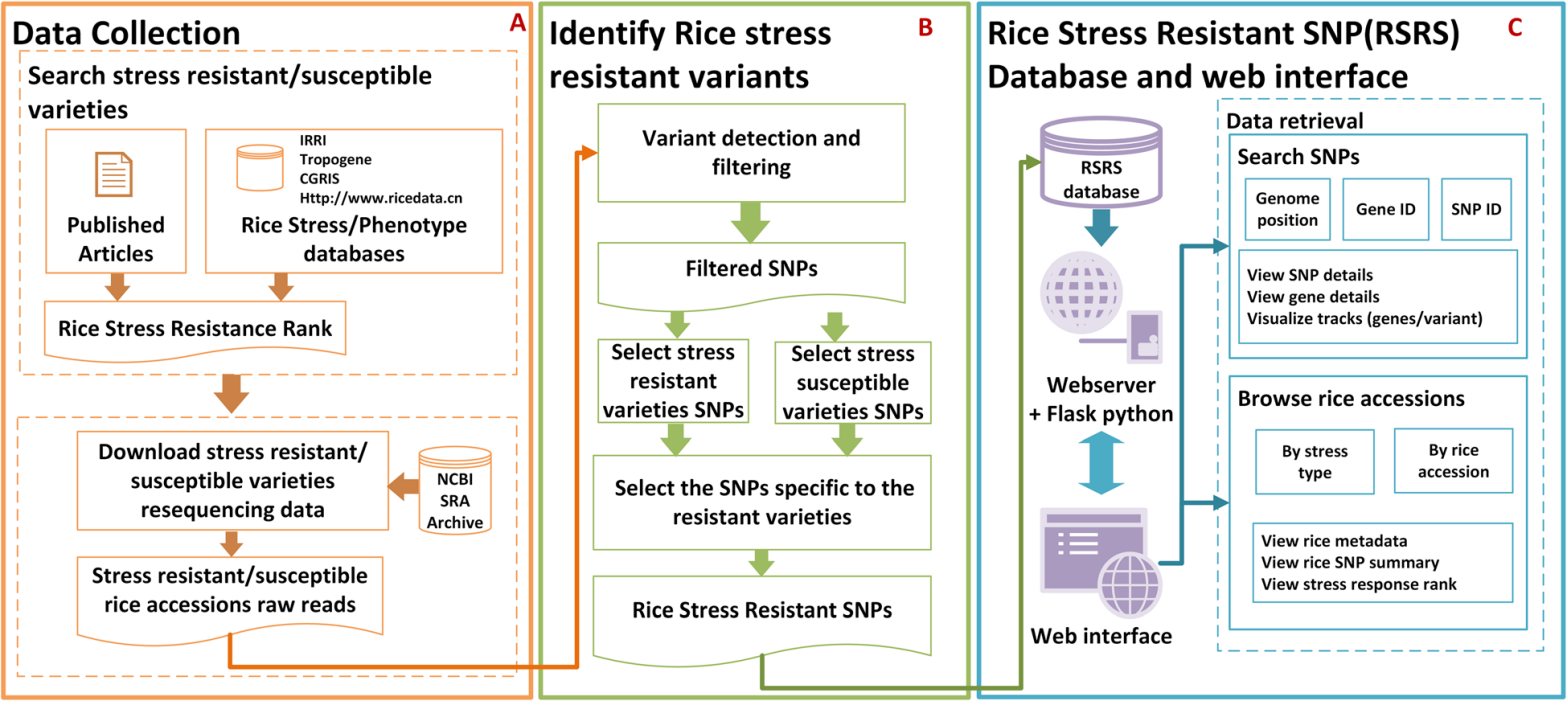
System view of RSRS database (**a**) Data collection of rice stress resistant/susceptible varieties and their re-sequencing data (**b**) Variant detection of the rice stress resistant/susceptible varieties and identification of the stress resistant specific SNPs (**c**) The RSRS database and web interface design layout

To compile the rice stress-resistant SNPs dataset, we first collected the stress resistant/susceptible rice varieties and their genomic re-sequencing data from previously conducted studies and publications (Fig. 1a). In this study, we collected the biotic stress- (rice blast fungus, bacteria, and pest) or abiotic stress- (heat, cold, flood, salt, alkali and zinc) resistance rank values for different varieties. The stress-responsiveness of each variety is ranked as Highly Resistant (HR), Resistant (R), Moderately Resistant (MR), HS (Highly Susceptible), Moderately Susceptible (MS) and Susceptible (S).

To provide a coherent view for the users, the RSRS database was organized on the basis of different stress types. Under each stress type, the list of stress-resistant rice varieties, metadata and their resistance rank was provided. The corresponding SNPs were also categorized under each stress group so that each SNP site in different rice varieties could be presented with its allelic frequency, annotation, the associated gene and GWAS supporting data if available.

### SNP Calling and Annotation

The variant calling pipeline followed the best practices of SNPs and Insertion/deletion (indel) discovery using Genomic Analysis Toolkit (GATK) v3.8 (Van der Auwera et al. 2013). This pipeline accepts genomic re-sequencing paired-end reads in SRA or FASTQ formats. The processing workflow was created using the snakemake workflow system (Köster and Rahmann 2012).

The quality check for the re-sequencing data of all the tested rice varieties was first conducted and the low-quality reads were trimmed using the Fastp trimming tool (Chen et al. 2018). After trimming, the clean reads were aligned to the rice reference genome (Nipponbare, IRGSP 1.0) (Kawahara et al. 2013) with Burrows-Wheeler Aligner (BWA) (Li and Durbin 2009) and indexed with SAMtools (Li et al. 2009). The aligned reads were sorted using Picard SortSam tool. Samples of each variety were merged using the SAMtools merge tool. The merged reads were deduplicated and read groups were added using the Picard MarkDuplicates and AddOrReplaceReadGroups tools, respectively (Broad Institute 2019). Subsequently, the GATK tools were used to recalibrate the base quality scores to obtain more accurate quality scores for each base.

Variant calling was then performed with a minimum Phred-scaled confidence threshold of 30 using the GATK-HaplotypeCaller (Van der Auwera et al. 2013) in GVCF mode to emit a GVCF file of each variant, and GATK-GenotypeGVCFs tool genotyped the variant files with the multi-sample model. Finally, the raw variants generated from the variant calling steps were further filtered to identify the high quality variants by using the GATK-VariantFiltration tool with the GATK recommended filtering criteria, QD < 2.0, FS > 60.0, MQ < 40.0, MQRankSum < − 12.5 and ReadPosRankSum < − 8.0, SOR > 4.0 (Van der Auwera et al. 2013).

### Identification of Stress-Resistant SNPs in Rice

After detecting the SNPs of all the tested varieties, we screened the stress-resistant SNPs based on the approach introduced by Silva et al. (2012) and Li et al. (2017). First, we detected the SNPs from the resistant and susceptible rice varieties using the variant detection pipeline mentioned in the SNP calling and annotation section. Second, we filtered out all the variants with read depth less than 3. Third, we grouped the SNPs into two categories under each stress type, one was detected from the stress-resistant rice varieties and the other was from the susceptible rice varieties. Fourth, we selected all the variants, which were detected in three or more stress-susceptible rice varieties. Fifth, we filtered out all the common variants observed between the resistant and susceptible groups and selected the SNPs unique to the resistant varieties (Fig. 1b). Finally, the effect of each variant on its target gene was predicted and annotated using the SnpEff 4.3r program (Cingolani et al. 2012), the SNPs were then classified based on their genomic region and variant effect.

In this study, we mainly focused on non-synonymous SNPs (nsSNPs) and other major effect SNPs as they could lead to amino acid residue change and alter the functional or structural properties of the target protein. Furthermore, we classified the genes associated with non-synonymous variants based on their functional annotation using the agrigo online functional annotation toolkit (Tian et al. 2017). The Physical positions and annotations of the rice genes were retrieved from RAP (https://rapdb.dna.affrc.go.jp/) databases. The GO terms and assignments for rice genes were downloaded from Gramene database (http://www.gramene.org/). The RAP rice gene locus IDs in our database were converted to MSU IDs using the RAP-DB ID converter tool (http://rapdb.dna.affrc.go.jp/tools/converter).

### Database Design and Web-Application Architecture

Three software components, including an Apache webserver (https://httpd.apache.org/), MySQL 5.7 relational database (http://www.mysql.com/) and Flask web development library (http://flask.pocoo.org/), were used to construct the RSRS database server-side (Fig. 1c).

**Fig. 2:**
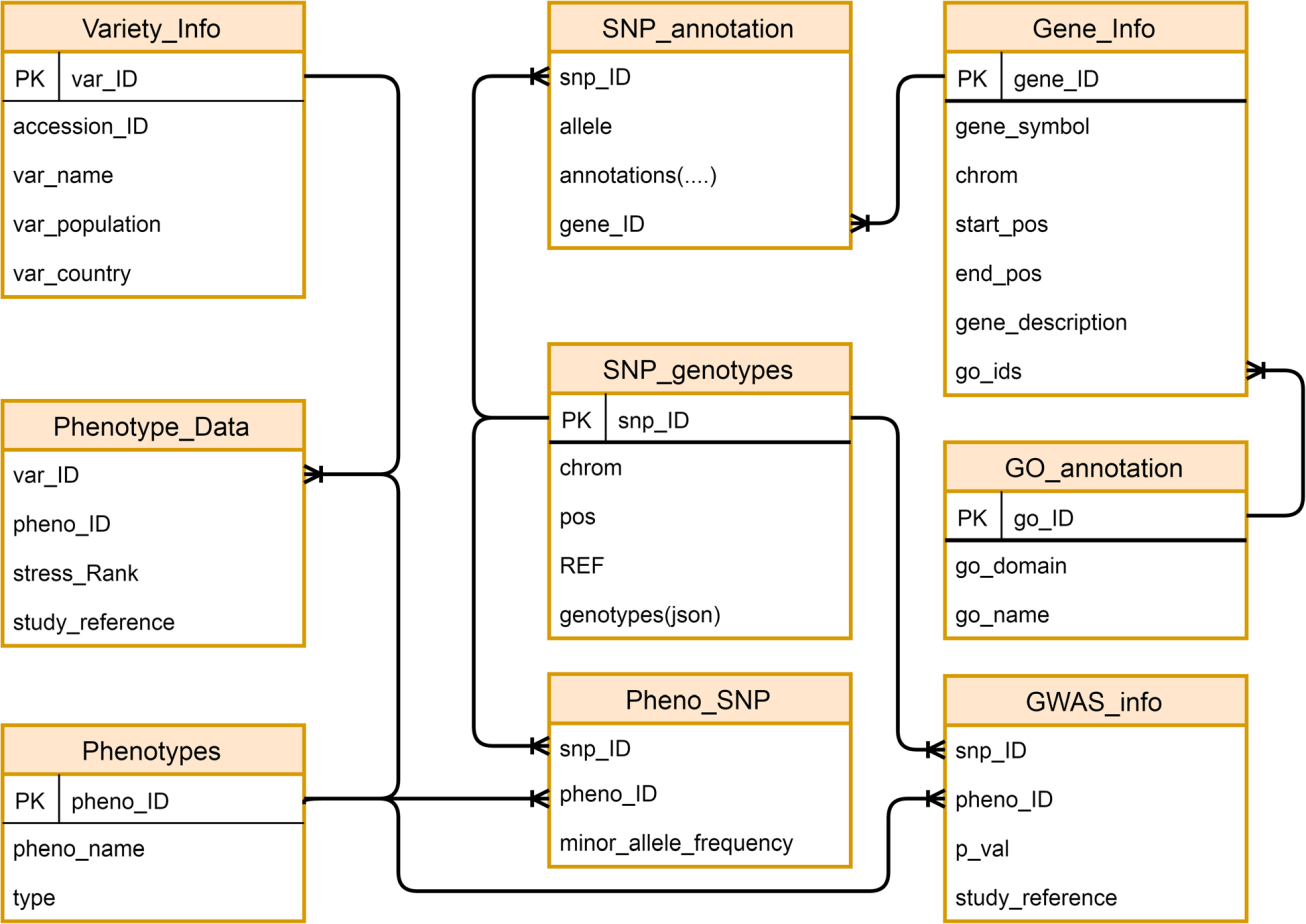
Entity-Relationship Diagram for the RSRS database. The Pheno_SNP table is the table, which connects the phenotype details with the genotype details. In this table the pheno_ID field is used to identify the associated stress with the varieties and the snp_ID is used to identify the associated SNPs with the varieties. The Pheno_SNP table have a 1:N relationship with the Phenotypes and SNP_genotypes tables using pheno_ID and snp_ID keys. The SNP_genotypes table describes the detail of each SNP, which is characterized by its own unique ID, snp_ID, chrom, position, reference allele and genotypes of each accession. A SNP may consist of multiple alleles in which the annotation of each allele is stored in the SNP annotation table. The SNP_annotation table is connected in a 1:N relationship with the Gene_info table, which stores the detail of each gene. The Gene_Info table is connected to the GO_annotation table to characterize the associated genes with each SNP. The variety and phenotype details are stored in Variety_Info, Phenotype_data and Phenotypes table, var_ID and pheno_ID are used to connect these tables. The GWAS_info table stores the associated GWAS record of each SNP with its associated stress. The ER diagram was created by using draw.io at https://www.draw.io/

In the RSRS database, nine MySQL tables were set up, including a ‘SNP_genotype’ table, a ‘SNP_annotation’ table, a ‘Gene_info’ table, a ‘GO_annotation’ table, a ‘GWAS_Info’ table, three phenotype data tables and a ‘Pheno_SNP’ table. The phenotype data tables stored the rice variety and stress related information, while the ‘Pheno_SNP’ table stored SNPs associated with each stress type. ‘var_ID’ uniquely identified each variety and ‘pheno_ID’ was defined to group the varieties resistant/susceptible to each stress type category. These two keys were used to link the phenotype data tables. The ‘SNP_genotype’ table stored the chrom, position, reference and genotypes of each SNP retrieved from the variant detection result files. Each SNP may have more than one allele. The annotation of each SNP allele is stored in the ‘SNP_annotation’ table. ‘SNPID’ was defined to identify each SNP uniquely and it is the key used to connect the SNP_genotype table with the SNP_annotation table. For efficient storage and data retrieval, the genotypes of each SNP were stored as JSON datatype. The ‘Gene_Info’ and ‘GO_annotation’ tables were used to store the detail of each gene and gene ontology annotations downloaded from the rice annotation database (https://rapdb.dna.affrc.go.jp/) and gramene database (http://www.gramene.org) respectively. The ‘gene_ID’ was defined as a primary key in ‘Gene_Info’ table, it linked this table to the SNP_annotation table to relate the associated genes with each SNP. The ‘GWAS_info’ table stored the GWAS records associated with the SNPs in each stress type. The entity-relationship (ER) diagram of the RSRS database is shown in Fig. 2.

RSRS web service was implemented in Python (https://www.python.org/) and based on the Flask web development library (http://flask.pocoo.org/). The Web Services consist of the phenotype and genotype data retrieval and provides the visualization tools to display the search results. The searchable terms include phenotype name, SNP ID, gene ID and chromosome region (Fig. 1c). The user’s query was passed from the front-end, the server initiated and displayed the output from the database module script. All the extracted information was displayed in a tabular format.

The interactive user interface was developed using the Bootstrap3 (http://www.getbootstrap.com) framework which combines HTML, CSS and JavaScript. Visualization of the data via statistical graphs and the tabular view was implemented using JavaScript charting libraries called Highcharts (https://www.highcharts.com/) and Datatables (https://datatables.net/), respectively. We hosted the system on a server running Ubuntu 16.04LTS powered by Apache server (Apache Version 2.4).

## Results and Discussion

### Database Content

The whole genome sequencing data of 402 rice (*Oryza sativa L.*) varieties with different abiotic and biotic stress-resistant ability were downloaded from the 3000 rice genome re-sequencing project and other projects in NCBI SRA database. Following the protocol introduced in Fig. 1, the abiotic and biotic stress-resistant rice varieties and their SNPs were detected and stored in the RSRS database.

#### Phenotype Data

The information for each variety including country of origin, ID, variety name and variety group was collected from the IRIC information site (http://iric.irri.org/resources/3000-genomes-project) and other related studies. The accession ID and the ID used for the variety is shown in the Additional file 1: Table S1. Additionally, we also collected the stress responsiveness of all the tested varieties from previous studies and phenotype databases. The list of the varieties in each stress group and the detailed information of each variety including the related references is also shown in the Additional file 1: Table S1.

##### SNP Data

**Fig. 3:**
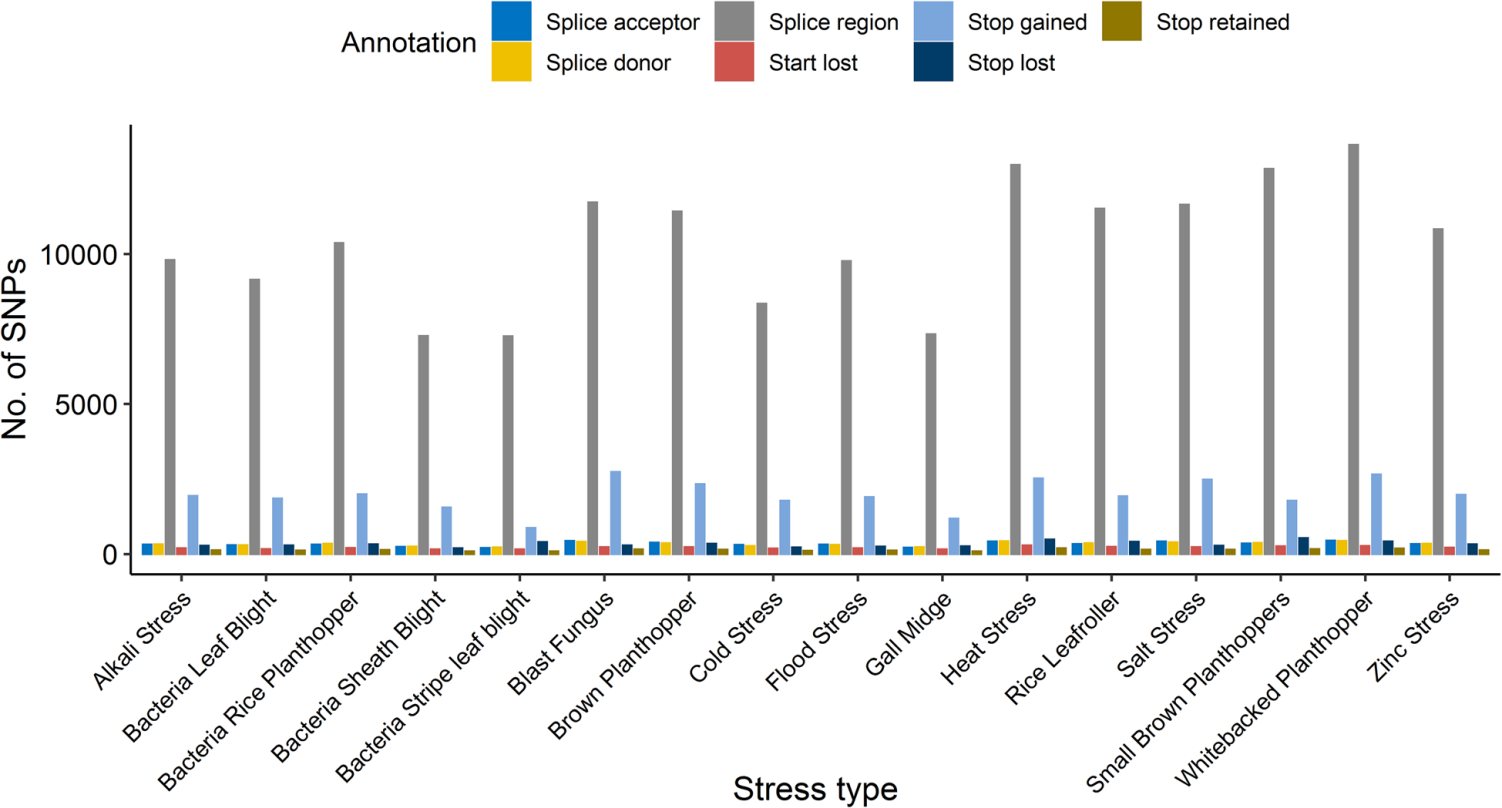
Number of abiotic and biotic stresses-resistant SNPs in splice region and other large effect SNPs. The large effect SNPs include start/stop gained, lost or retained, and splice donor and acceptor. Start and stop codon gained or lost SNP induce the gain or loss of the start/stop codon. SNP located in the stop codon may retain the stop codon function (Stop codon retained). The SNP generating a splice acceptor, donor or region are named splice acceptors, splice donor of splice region

After detecting the genetic variants in each tested rice variety genome and filtering the stress-resistant SNPs from the stress-resistant rice varieties, more than 9.5 million stress-resistant SNPs in rice were identified and stored in the RSRS database. Table 1 summarizes the number and effect of the stress-resistant SNPs located in the different genomic regions, including genic, intergenic, intronic and upstream/downstream regions. The stress-resistant SNPs located in particular positions, such as start and stop codons, splice donor and acceptor sites, are also displayed in Fig. 3.

**Table 1:** Number of stress-resistant SNPs located in different regions of rice genome

Stress types	3’UTR^b^	5’UTR^b^	Downstream^b^	Intergenic^b^	Intron^b^	CDS^a^	Upstream^b^
Heat Stress	93,767	55,759	653,058	635,265	63,162	205,078	2,780,114
Alkali Stress	66,920	37,644	388,645	331,550	43,425	147,100	1,755,014
Salt Stress	79,546	44,882	470,148	409,469	51,300	175,835	2,081,462
Flood Stress	67,419	37,926	391,338	334,562	43,764	142,223	1,787,105
Cold Stress	58,798	33,903	370,763	332,310	40,007	126,918	1,612,926
Zinc Stress	74,843	41,853	411,990	330,483	48,027	161,716	1,908,842
Blast Fungus	81,491	47,765	522,328	480,785	53,365	183,547	2,256,982
Bacteria Leaf Blight	63,649	35,647	368,539	319,163	41,456	138,399	1,633,392
Bacteria Sheath Blight	51,663	29,237	331,287	272,583	34,716	112,617	1,356,611
Bacterial Rice Planthopper	72,686	41,112	417,411	347,624	45,912	155,346	1,890,893
Bacteria Stripe leaf blight	49,221	27,107	269,081	203,861	31,815	103,938	1,274,279
Brown Planthopper Pest	79,293	44,994	475,951	410,522	52,299	173,293	2,109,723
Gall Midge Pest	50,495	29,177	291,121	230,150	32,704	107,667	1,367,183
Small Brown Planthoppers Pest	86,755	47,381	465,586	363,512	55,723	182,484	2,205,066
Whitebacked Planthopper Pest	94,541	53,730	552,635	462,375	61,037	203,404	2,508,144
Rice Leafroller Pest	79,704	44,658	448,760	358,452	50,581	172,603	2,072,717

^a^CDS includes the SNPs in exonic region including nsSNPs (non-synonymous SNPs) and sSNPs (synonymous SNPs)

^b^Intergenic, 5’UTR, Intron 3’UTR, Upstream and Downstream indicate that No. of stress-resistant SNPs located in the Intergenic, 5’UTR, Intronic, 3′ UTR, upstream and downstream regions of a gene

### Biotic Stress-Resistant SNPs

In the biotic stress-resistant SNPs category, there are three sub-group SNPs, which are resistant to 3 kinds of biotic stresses, including fungus, bacteria and pest infection.

#### Blast Fungus-Resistant SNPs

A final set of 6,785,349 SNPs were called from 152 blast-resistant rice varieties using Nipponbare as the reference genome. We detected 3,324,526 SNPs from 26 highly blast-susceptible rice varieties using the same pipeline. After screening the SNPs specific to the blast-resistant varieties, 3,638,230 potential blast-resistant SNPs were detected and stored in this sub-group.

**Fig. 4:**
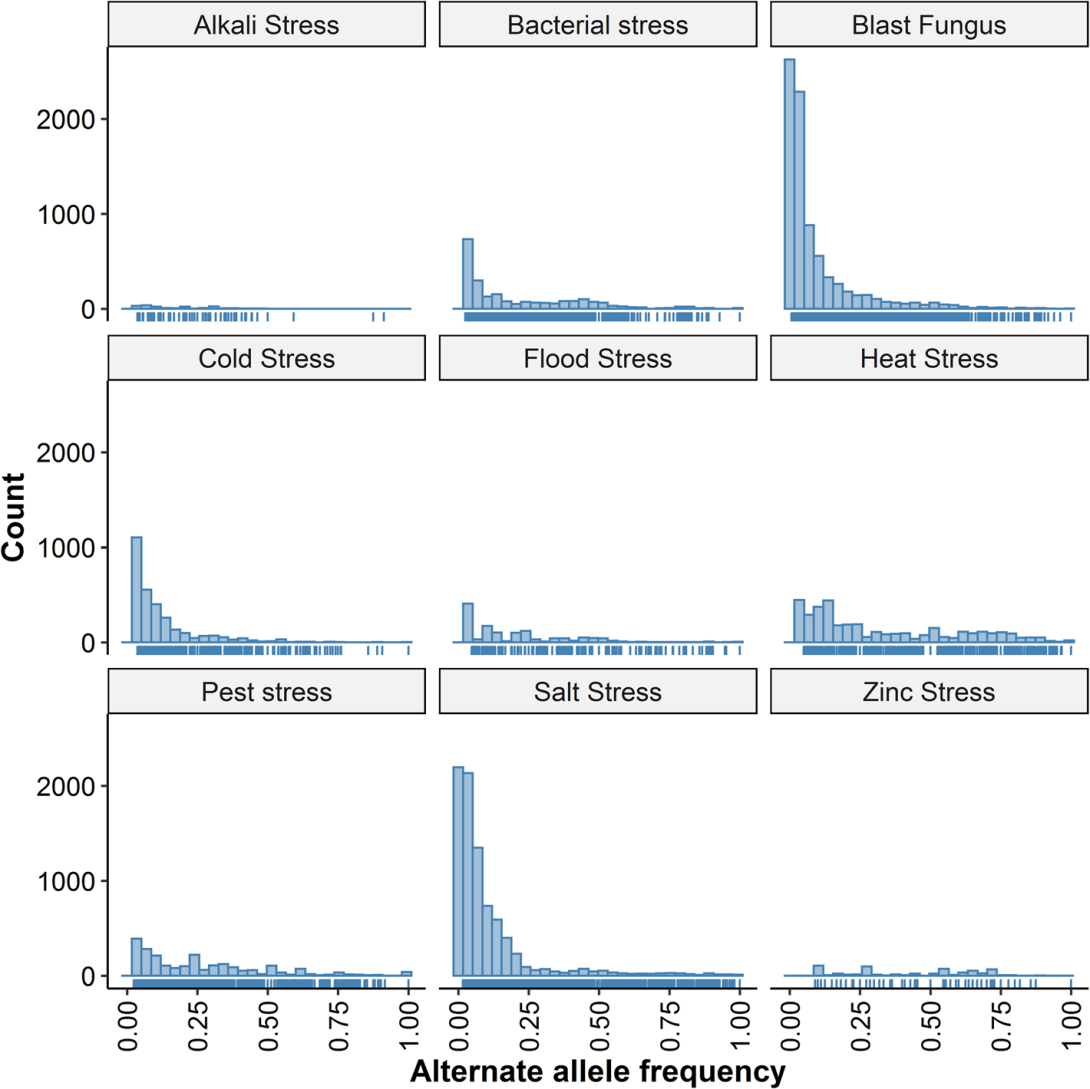
The variants distribution by annotation in different abiotic and biotic stress-resistant genes in rice. The X-axis represents the variant annotation of the SNPs in these genes and the Y-axis represents the count of each annotation in these genes

**Fig. 5:**
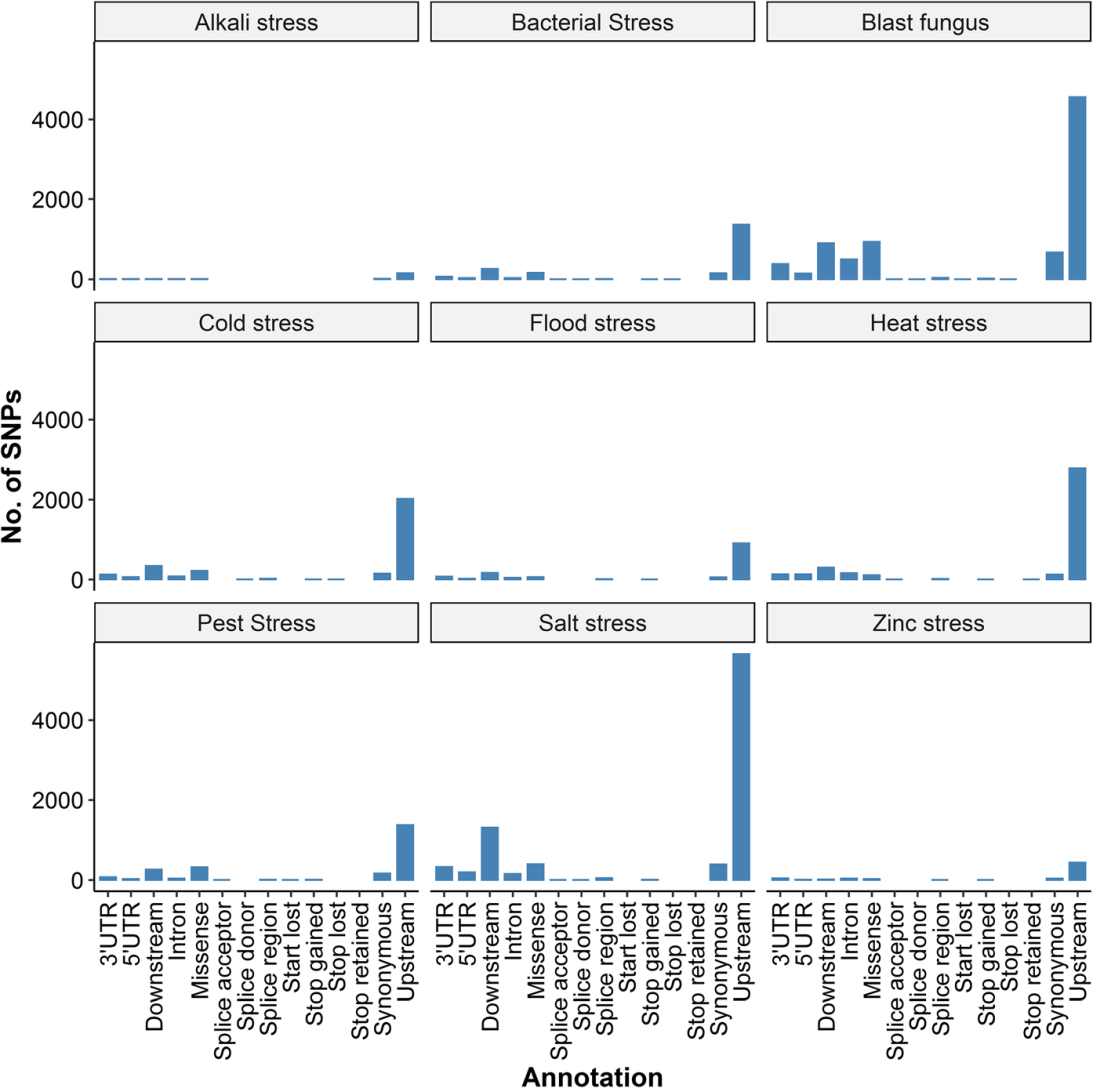
The alternate allelic frequency distribution in different abiotic and biotic stress-resistant genes in rice. The X axis represents the allele frequencies of the non-reference alleles and the Y axis represents of the count of each allele frequency of the SNPs in these genes

In these blast-resistant rice varieties, we identified 24,352 genes harboring blast-resistant nsSNPs, which were regarded as potential blast-resistant genes in rice. Based on the functional annotation of these genes, 609 of the potential blast-resistant genes were associated with stress stimuli and response ability. Interestingly, 139 of them had been reported to function as blast-resistant genes in different references, which were shown in Additional file 2: Table S2. Among the 139 known blast-resistant genes, 24 genes were transcription factors, 34 genes were disease resistance genes, and the other 81 genes were involved in different families of stress-response abilities. In total, there were 8005 stress-resistant SNPs on these 139 genes, in which 11.5% were missense, 67.0% were in the upstream/downstream region, 8.6% were synonymous variants and 12.0% include the others (Fig. 5). These variants were distributed in the rice varieties with allele frequency between 0.01 and 1.0 (Fig. 4).

#### Bacterial Stress-Resistant SNPs

We first collected 74 bacterial-resistant rice varieties, including 41 bacterial leaf blight-resistant varieties with a total of 4,307,743 SNPs, 18 bacteria sheath blight-resistant varieties with a total of 3,991,441 SNPs, 5 bacteria stripe leaf blight resistant varieties with a total of 1,971,323 SNPs and 24 bacteria planthopper-resistant varieties with a total of 4,242,547 SNPs. Similarly, we had 8 bacterial leaf blight-susceptible varieties with 1,856,574 SNPs, 7 bacterial sheath blight-susceptible varieties with 1,999,162 SNPs, 6 bacterial rice planthopper-susceptible varieties with 1,344,685 SNPs and 3 bacteria stripe leaf blight-susceptible varieties with a total of 523,829 SNPs. Finally, we generated 2,608,978, 2,176,113, 2,980,714 and 1,965,432 SNPs resistant to bacterial leaf blight, sheath blight, rice planthopper and stripe leaf blight in the rice genome, respectively.

We identified 19,154, 17,711, 20,972 and 17,987 genes harbouring bacterial leaf blight, sheath blight, rice planthopper, and stripe leaf blight-resistant nsSNPs, respectively. Among them 483, 433, 518 and 447 were associated with stress stimuli and responses based on the functional annotation. Interestingly, 64 bacterial leaf blight, 11 bacterial sheath blight, 22 bacterial rice planthopper and 7 rice stripe disease-resistant potential genes had been confirmed in different publications, shown in Additional file 2: Table S2. These genes included 15 transcription factors, 19 disease resistance genes and others associated with defence response activities. The bacterial stress-resistant SNPs distributed on these genes consist of 7.43% nsSNPs, 6.78% synonymous SNPs and 79.5% upstream/downstream (Fig. 5). The allelic frequency distribution of the SNPs in these genes varies between 0.022 and 1.0 (Fig. 4). Additionally, (Zhang et al. 2017) verified 121 significantly enriched SNPs which contributed to bacterial leaf blight-resistance in rice. Among them, 91 were mapped to the bacterial leaf blight-resistant SNPs in the RSRS database. In another research work, 46 SNPs were found to be associated with bacterial blight-resistance in rice (Dilla-Ermita et al. 2017), among which 29 were also detected in the RSRS database.

#### Pest Stress-Resistant SNPs

A total of 81 pest-resistant rice varieties were identified, including 45 brown planthopper pest-resistant varieties with 6,054,118 SNPs, 4 gall midge pest-resistant varieties with 3,638,406 SNPs, 8 small brown planthopper pest-resistant varieties with 4,221,428 SNPs, 35 white backed planthopper pest-resistant varieties with 5,957,813 SNPs and 10 rice leafroller pest-resistant varieties with 4,679,556 SNPs. Similarly, we detected 3,710,640 SNPs from 8 brown planthopper-susceptible rice varieties, 4,637,279 SNPs from 3 gall midge susceptible varieties, 2,392,253 SNPs from 3 small brown planthopper-susceptible varieties, 3,362,269 SNPs from 6 white backed planthopper-susceptible varieties, and 3,055,842 SNPs from 3 leaf roller pests-susceptible varieties. Finally, we found 3,357,284 brown planthopper, 2,115,252 gall midge, 3,417,213 small brown-, 3,948,989 white backed planthopper- and 3,237,416 rice leafroller pest-resistant SNPs in rice, respectively.

In the pest-resistant varieties, we identified a total of 22,876, 19,782, 23,721, 24,098, 22,101 genes harboring nsSNPs in brown planthopper pest, gall midge pest, small-brown planthopper pest, white-backed planthopper pest and rice leafroller pest-resistant rice varieties respectively. We classified these genes based on their functional annotation and we found that 555, 489, 614, 603 and 540 genes were associated with stress and stimuli response related functions. Among them, 29 genes have been reported to be involved in pest caused diseases resistance, which is shown in the Additional file 2: Table S2. These genes were constituted of 3 transcription factors, 14 pathogen resistance genes and other defence related functions. The total number of SNPs detected in these genes included 72.1% of upstream/downstream, 14% of nsSNPs and 7.08 of synonymous SNPs (Fig. 4). The allelic diversity of the SNPs varies within the range of 0.022 to 1 (Fig. 5).

### Abiotic Stress-Resistant SNPs

In the abiotic stress category, we detected SNPs in the genome of rice varieties with resistant ability to heat, alkali, salt, cold, flood and zinc stresses.

#### Heat Stress-Resistant SNPs

Twenty heat stress-resistant rice varieties were used in this study and a total of 5,707,672 SNPs were detected from this set of rice varieties. Similarly, we detected 1,233,652 SNPs from 8 highly heat-susceptible rice varieties. Finally, a total of 4,500,192 heat stress-resistant specific SNPs in rice were identified.

In the genome of heat stress-resistant rice varieties, we identified a total of 26,357 genes harbouring heat stress-resistant nsSNPs. Based on the functional annotation, 661 genes of them were associated with stress response and stimuli. Interestingly, 75 genes had been reported to be involved in heat stress tolerance in rice. Among these 75 genes, 6 genes were transcription factors and 23 are heat shock proteins, which are shown in the Additional file 2: Table S2. The total number of SNPs in these genes was 3745, including 82% upstream/downstream, 3% nsSNPs, 3.2% synonymous and 12% other variants. The allele frequency of these variants varied between 0.047 and 1 (Figs. 4 and 5).

#### Alkali Stress-Resistant SNPs

Twenty-seven alkali stress-resistant rice varieties were used in this study. The total number of SNPs called from this group was 4,931,032. From the 10 susceptible rice varieties, we detected 2,314,097 SNPs. After filtering out the common variants between the resistant and susceptible varieties, we generated 2,779,427 alkali stress-resistant SNPs in rice.

In this subcategory, we identified a total of 22,876 genes harbouring alkali stress-resistant nsSNPs. Based on the functional annotation, 513 genes were associated with stress response and stimuli. Among them, five genes have been reported to be associated with alkali stress tolerance in rice, which is shown in the Additional file 2: Table S2. The total number of SNPs associated with these genes was 182 and the allele frequency varies between 0.022 and 0.9 (Figs. 4 and 5).

#### Salt Stress-Resistant SNPs

Sixty-one salt stress-resistant rice varieties were used in this study. The number of SNPs identified from this set of rice varieties was 6,080,805. Similarly, we detected 2,924,352 from 17 salt stress-susceptible rice varieties. After filtering out the SNPs in salt stress-susceptible rice varieties, we identified a total of 3,323,770 salt stress-resistant specific SNPs in the salt stress-resistant varieties.

In these salt-stress resistant varieties, we mapped a total of 23,461 genes harbouring salt stress-resistant nsSNPs. Based on the functional annotation, 589 genes were associated with stress response and stimuli. Interestingly, 209 genes have been reported to be associated with salt-tolerance in rice, including 30 transcription factors, 26 kinase family genes, and 14 ion transporter family, which are also shown in Additional file 2: Table S2. There were a total of 8472 SNPs in these genes, including 4.65% of missense, 81.4% of upstream and downstream, 4.6% of synonymous variants and others (Fig. 5). The allele frequency of these variants varied between 0.012 and 1 (Fig. 4).

In a study conducted by Lekklar et al. (2019), they found 448 significantly enriched SNPs contributing to salt stress-resistant ability in rice by using Genome-Wide Association Study (GWAS) method, among which 389 were also detected in the results of this study and 162 were stored as salt stress-resistant SNPs in the RSRS database. Additionally, Jain et al. (2014) screened 910 SNPs present in the coding region of 346 differentially expressed genes from 3 salt stress-resistant rice varieties, of which 862 were consistent with the results of this study and 301 were stored as salt stress-resistant SNPs in the RSRS database.

#### Cold Stress-Resistant SNPs

Twenty-seven cold stress-resistant rice varieties were collected in this study, in which we detected a total number of 4,906,148 SNPs. From 10 cold-susceptible rice varieties, we detected a total of 2,560,928 SNPs. Finally, we filtered a total of 2,584,211 cold stress-resistant SNPs in rice.

We mapped 16,217 genes with cold stress-resistant nsSNPs in rice. Based on the functional annotation, 476 genes were associated with stress response and stimuli. Interestingly, 107 genes have been reported to be associated with cold tolerance in rice, which is shown in Additional file 2: Table S2. There were a total of 2998 SNPs located in these genes, in which 88% were upstream/downstream, 7.2% were nsSNPs and 4.84% were synonymous variants (Fig. 5). Their allelic frequency variation ranged from 0.038 to 1.0 (Fig. 4). Furthermore, the data in other publications also supported our results. Wang et al. (2016) found 181 significantly enriched cold stress-related SNPs in rice of which 169 were consistent with the SNPs in the RSRS database (Wang et al. 2016).

#### Flood Stress-Resistant SNPs

Twenty-one flood stress-resistant rice varieties with a total of 4,851,632 SNPs and 10 flood stress-susceptible rice varieties with 2,238,878 SNPs were collected in this study. Finally, we identified 2,813,598 flood stress-resistant SNPs in flood stress-resistant rice varieties.

In these flood stress-resistant varieties, we identified a total of 20,818 genes with flood stress-resistant nsSNPs. Based on the functional annotation, 505 genes were associated with stress response and stimuli. Twenty-six of the genes, so far, have been reported to be associated with submergence tolerance, including five transcription factors and five ethylene receptor like proteins, which are also shown in Additional file 2: Table S2. In these 26 genes, we detected 1321 SNPs, of which 80.0% were upstream/downstream SNPs, 4.47% were nsSNPs and 4.1% were synonymous SNPs.

#### Zinc Stress-Resistant SNPs

A total of 4,215,431 SNPs were detected in 11 zinc stress-resistant rice varieties, while 1,345,257 SNPs were found in the five zinc stress-susceptible rice varieties. In total 2,987,375 zinc stress-resistant SNPs in rice were identified from the zinc stress-resistant rice varieties.

In these zinc stress-resistant varieties, we identified 20,818 genes with zinc stress-resistant nsSNPs. Based on the functional analysis, 530 of the genes were associated with stress response and stimuli. Six genes, so far, have been reported to be involved in zinc stress tolerance, which are mainly involved in Zn uptake and translocation as shown in the Additional file 2: Table S2. In these six genes, there were 538 SNPs, including 74% upstream/downstream, 4% nsSNPs, and 6% Synonymous SNPs (Fig. 5). The allele frequency of these SNPs varied between 0.047 and 0.89 (Fig. 4).

### Web Interface

RSRS is an integrated rice stress resistant SNP database allowing users to explore overall data organization, to obtain information including rice stress related information, stress resistant SNPs and associated genes by querying a specific genomic region, gene ID or SNP ID, and to visualize the data of interest in different output format. The functions of RSRS database were shown in Fig. 1c.

RSRS database allows users to search, compare and browse the phenotypic and genotypic data of the stress-resistant rice varieties used in our study. Each rice variety in the RSRS database is assigned with a cultivar ID. The variety browse function presents the phenotype detail of each rice cultivar including the geographical information, population, and the stress-resistance rank. The SNP browse function provides users to bring up the overall SNP summary in each stress-resistant group, SNP summary per chromosome in each cultivar and the distribution of different type of SNPs in each group.

**Fig. 6:**
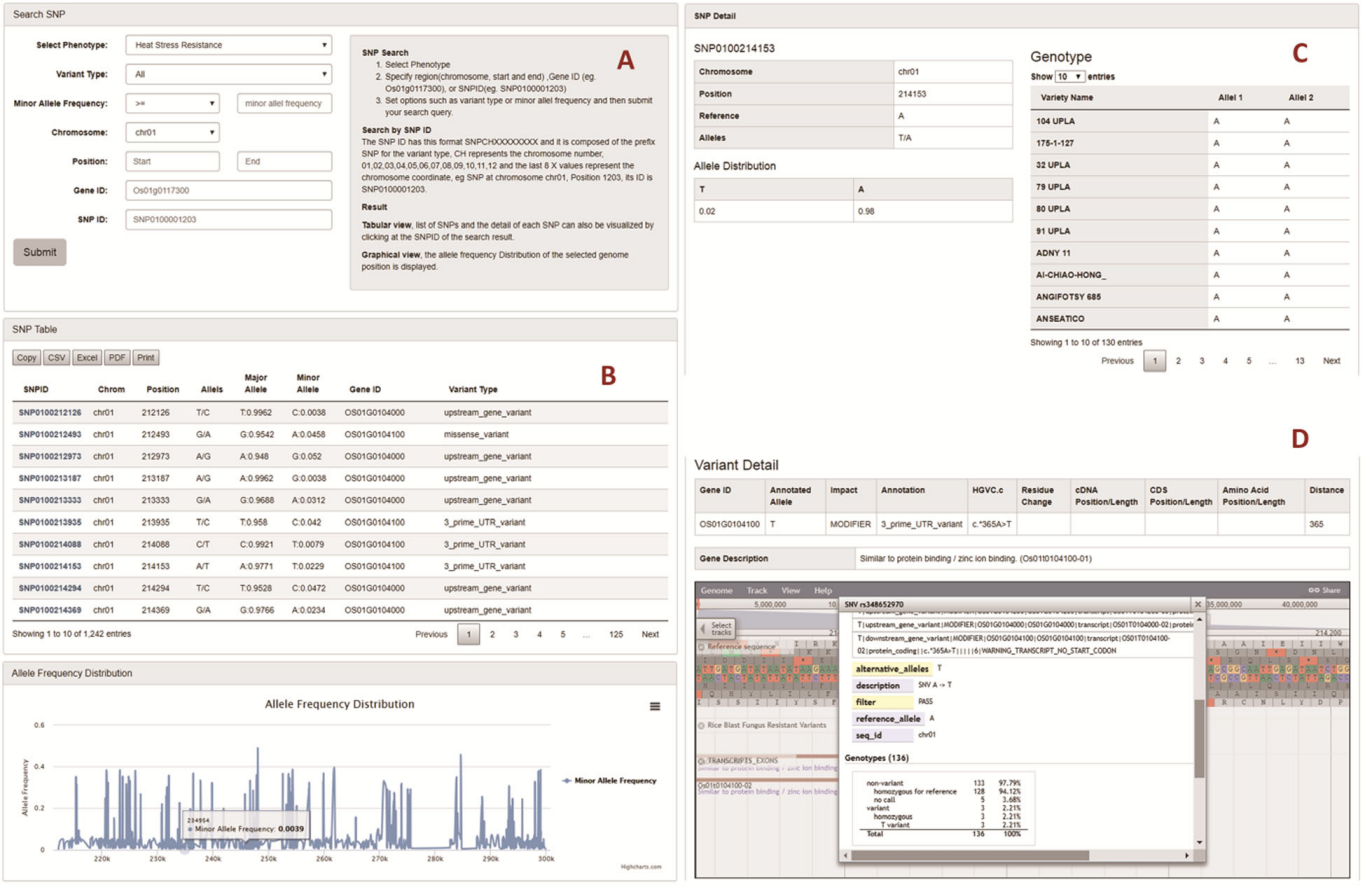
Examples of search functions in RSRS database. Search Interface (**a**) allows users to search by region, gene ID and SNP ID. Additionally users can also set options, such as allele frequency, variant types. Search Results (**b**) retrieves the list of SNPs and displays the allele distribution in a given range. SNP detail view (**c**) displays the detail of each SNP and the genotype of each rice accession. Jbrowse visualization (**d**) displays the SNP detail in Jbrowse and gives the detail of the associated gene with the SNPs

The searching function is a user-friendly web interface for users to query SNP related information. The users are able to search for different SNPs by a given SNP ID, gene ID, phenotype, variant annotation, chromosome or a specific genomic region (Fig. 6a).

Searching by genomic region allows users to search for SNPs at a selected chromosome in a given range of genome position. Besides this, retrieval of SNPs using different search options like variant annotation, minor allele frequency can be facilitated in the database.

Searching by SNP ID allows users to search for a specific SNP by using its ID. Each SNP has a unique identifier with a format of SNPCHXXXXXXXX. The first letter of the ID indicates the variant type, such as ‘SNP’ standing for SNP. The ‘CH’ stands for the chromosome number (01–12) and XXXXXXXX represents chromosome coordinate of a variation.

Searching by gene ID allows the user to search for SNPs associated with a given gene. Similarly, the user can also set the options to search for the variants of a selected annotation or range of minor allele frequencies (Fig. 6a).

The search function results (Fig. 6b) can also be further expanded and the detail of each SNP can be explored. The detail of each SNP includes the chromosome position, the allele distribution, the associated gene information, the variant effects annotation and the genotype of the SNP in each cultivar of the selected phenotype. This function helps the user to figure out the polymorphic SNPs between two or more rice cultivars (Fig. 6c). Additionally, we incorporated the GWAS enrichment scores of the SNPs with published data in this database. The SNPs with their available enrichment score will be displayed in the SNP detail page.

Furthermore, the RSRS database system also integrates the genome browser tool, JBrowse, to navigate the data in a graphical visualization format (Buels et al. 2016). JBrowse allows the users to directly visualize the location of SNPs, display the complete list of annotations, variant details, and genotype data of each accession. The interactive and dynamic genome browser could also display genomic features for each rice variety, gene and the corresponding data tracks, as well as variant alignment (Fig. 6d).

## Conclusion

The initial purpose of the RSRS database provides a convenient way to search and retrieve biotic and abiotic stress-resistant SNPs and their annotation for the rice research community. The RSRS database is built on the genomic re-sequencing data of different rice cultivars with different stresses-resistant ability. In total, over 9.5 million stress-resistant SNPs and 797 genes associated with stress response and stimulus were detected from more than 400 stress-resistant rice varieties. Interestingly, our results were partially supported by previous publications, indicating that the results in this study would give valuable insights for the researchers seeking to identify novel stress-resistant genes in rice.

We organized and presented the stress-resistant SNPs with related data, including genotype and phenotype, in a web-based system. By integrating with comprehensive information, this platform is growing to be a useful tool for a wide range of applications in rice genetics, breeding, and comparative genomics. This database will facilitate the researchers to investigate the genetic variation of the stress-resistant genetic variants in rice in a more streamlined, rapid, and efficient fashion.

We anticipate extending the services of this RSRS database by including more rice accessions with different stress-resistant capability as well as more kinds of stresses. Additionally, we will also make it more comprehensive knowledgebase for rice abiotic and biotic stress-related studies by incorporating other omics data, including transcriptome, proteomics, and metabolomics. We will also endeavour to make the database more user-friendly and more efficient with the suggestion from rice researchers and breeders who make use of this first version of RSRS database.

## Supplementary information

**Additional file 1: Table S1.** List of Rice Accessions Resistant/Susceptible to Different Biotic Stresses.

**Additional file 2: Table S2.** List of Genes Involved in Different Biotic/Abiotic Stress Resistance.

## Data Availability

RSRS database is freely available at http://bioinformatics.fafu.edu.cn/RSRS. Additionally, the data that support the findings of this study are also provided as supplementary material online.

## Abbreviations

GWAS: Genome-Wide Association Study
HR: Highly Resistant
HS: Highly Susceptible
JSON: JavaScript Object Notation
MR: Moderately Resistant
MS: Moderately Susceptible
nsSNP: Non synonymous SNP
QTL: Quantitative Trait Locus
R: Resistant
RSRS: Rice Stress Resistant SNPs
S: Susceptible
SNP: Single Nucleotide Polymorphism
WSGI: Web server gateway interface
